# Safe, Smart, and Scalable: A Prospective Multicenter Study on Low-Dose CT and CTSS for Emergency Risk Stratification in COVID-19

**DOI:** 10.3390/jcm14134423

**Published:** 2025-06-21

**Authors:** Andrzej Górecki, Piotr Piech, Anna Bronikowska, Zuzanna Szostak, Ada Jankowska, Karolina Kołodziejczyk, Bartosz Borowski, Grzegorz Staśkiewicz

**Affiliations:** 1 Medical Diagnostic Center Voxel, Regional Hospital in Łańcut, 37-100 Łańcut, Poland; agorecki@mp.pl; 2 Department of Correct, Clinical and Imaging Anatomy, Medical University of Lublin, 20-090 Lublin, Poland

**Keywords:** COVID-19, low-dose CT, chest CT severity score, emergency medicine, risk stratification, prospective study

## Abstract

**Background:** Effective early risk stratification in COVID-19 remains a critical challenge in emergency care, particularly due to the limitations of RT-PCR testing, including delayed processing and false negatives. There is an unmet need for imaging tools that are fast, reliable, and safe for repeated use in acute clinical settings. **Methods:** In this prospective, multicenter study, over 1000 patients hospitalized with suspected or confirmed COVID-19 were initially screened. A total of 555 patients with PCR-confirmed infection were ultimately included for analysis. All participants underwent low-dose chest CT (LDCT) at admission. Pulmonary involvement was assessed using the chest CT severity score (CTSS) based on a unified protocol. CTSS values were analyzed in relation to ICU admission, in-hospital mortality, demographic data, oxygen saturation, dyspnea scores, and laboratory markers (CRP, LDH, lymphocyte, and neutrophil counts). Imaging was interpreted by board-certified radiologists under harmonized reporting standards. **Results:** CTSS values ≥13 and ≥15 were significantly associated with ICU admission and in-hospital mortality, respectively (*p* < 0.01). Strong correlations were observed between the CTSS and CRP, LDH, and dyspnea scores, with negative correlations to oxygen saturation and lymphocyte count. The standardized LDCT protocol ensured consistent image quality and minimized radiation exposure. **Conclusions:** LDCT combined with the CTSS provides a robust, reproducible, and radiation-sparing method for emergency risk stratification in COVID-19. Its high clinical utility supports deployment in frontline triage systems and future AI-enhanced diagnostic workflows.

## 1. Introduction

Severe Acute Respiratory Syndrome Coronavirus-2 (SARS-CoV-2) was first identified in China in December 2019 and swiftly became a global health concern. The rapid spread of the virus across all continents led the World Health Organization to declare a pandemic on 11 March 2020 [1]. By July 2020, there had already been 14 million confirmed infections and 582,000 deaths [2]. The incubation period for SARS-CoV-2 averages 6.4 days. It spreads predominantly via droplet transmission, leading to clinical manifestations typical of respiratory tract infections, such as fever, cough with accompanying dyspnea, and muscle pains. Some patients experience severe hypoxia requiring hospitalization and mechanical ventilation [1]. The COVID-19 pandemic rapidly tested the resilience of healthcare systems worldwide, leading to substantial treatment expenses. In the U.S., the average hospitalization cost per SARS-CoV-2-infected patient amounted to USD 14,366, whereas non-hospitalized infected individuals incurred costs of around USD 3045 [3].

Real-time reverse transcription-polymerase chain reaction (RT-PCR) remains the most widely used method for detecting SARS-CoV-2, though it has certain limitations potentially impacting diagnostic efficacy. The sensitivity of RT-PCR tests, compared to other diagnostic methods, is only about 60–70%, increasing the risk of false-negative results [4]. Therefore, imaging modalities have assumed a significant role in COVID-19 diagnostics, being recommended by various health organizations as complementary to conventional diagnostic methods [5]. Frequent presentations of pneumonia-like symptoms among patients further substantiate the validity of imaging techniques, enabling evaluation of the progression and complications of the disease process.

Initially, computed tomography (CT) was widely utilized, illustrating pathological pulmonary changes such as ground-glass opacities (GGOs), air bronchograms, and interlobular septal thickening [6]. However, concerns regarding excessive patient exposure to ionizing radiation prompted research into equally effective yet safer imaging methods [7]. Low-dose computed tomography (LDCT) emerged as a promising alternative, significantly reducing the CTDlvol [mGy] and effective dose [mSv] compared to standard CT. Due to reduced radiation exposure, LDCT examinations can be performed repeatedly in individual patients for follow-up assessments. Additionally, studies indicate that LDCT reduces motion artifacts in pulmonary parenchymal imaging relative to standard CT [8].

**Figure 1:**
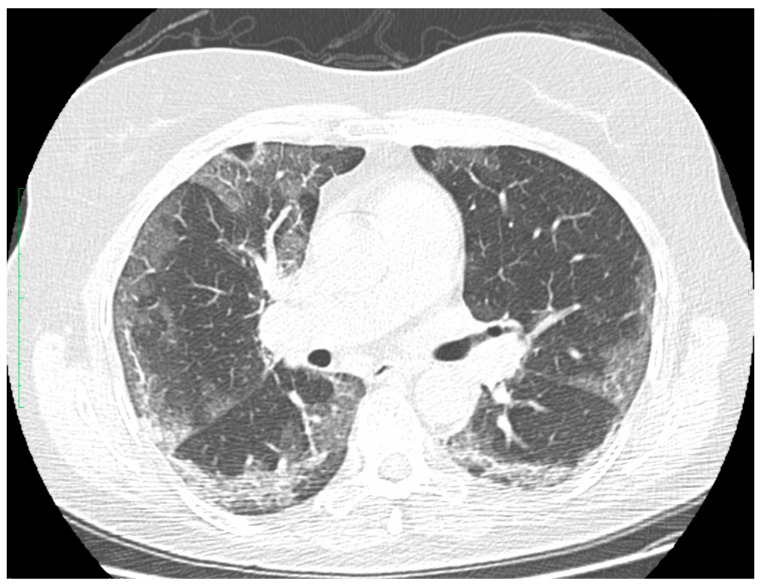
Axial low-dose chest CT image of a 61-year-old patient showing bilateral and peripheral ground-glass opacities.

**Figure 2:**
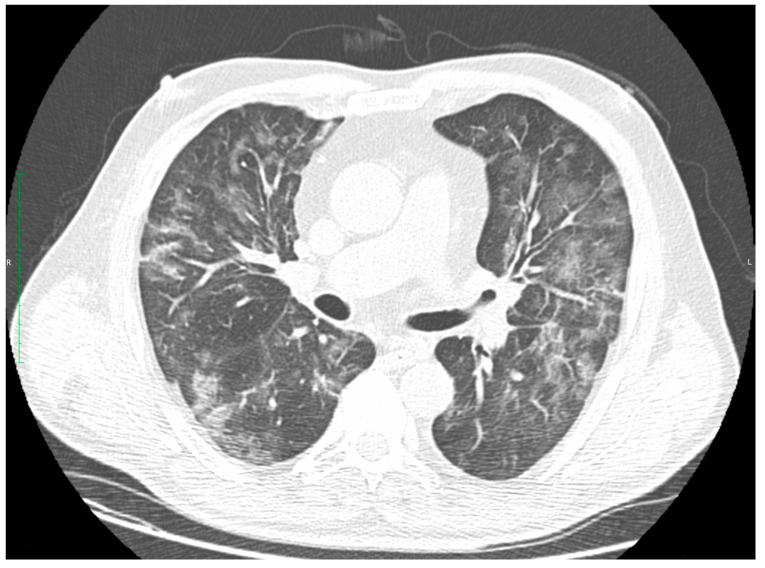
Axial low-dose chest CT image of a 67-year-old patient demonstrating extensive bilateral ground-glass opacities and consolidations.

The implementation of LDCT in diagnostics facilitated the development of the chest CT severity score (CTSS), used for evaluating pulmonary parenchymal involvement across each of the five lung lobes. Insights from conducted studies may prove critical in future diagnostic and therapeutic strategies adapting to emerging challenges associated with infectious disease progression. Examples of chest CT scans in patients with varying degrees of pulmonary involvement are shown in Figure 1 and Figure 2.

## 2. Materials and Methods

### 2.1. Study Objective

The objective of this study was to analyze the efficacy and safety of LDCT for monitoring the dynamics of pulmonary changes during COVID-19 among a large patient population and to derive an inflammatory change severity index for LDCT—CTSS which defines observation endpoints such as hospitalization duration, ICU treatment necessity, or mortality risk.

### 2.2. Study Design and Ethical Approvals

The project was conducted according to best practices and Frascatti manual guidelines between 27 October 2020 and 26 January 2022 and funded by the National Centre for Research and Development. The study received positive approval from the Bioethics Committee at the University of Rzeszów. It is worth highlighting that 100% of the literature cited in this article was published within the last 5 years, further strengthening the credibility of the obtained results.

### 2.3. Participant Characteristics

**Figure 3:**
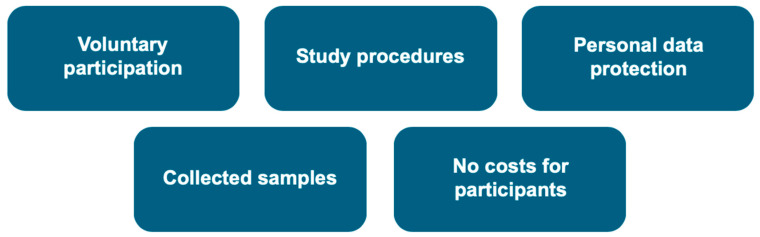
Key components of the patient information form provided upon hospital admission.

A group of 1000 patients was presented with an original informational form, the content of which is depicted in Figure 3.

**Scheme 1:**
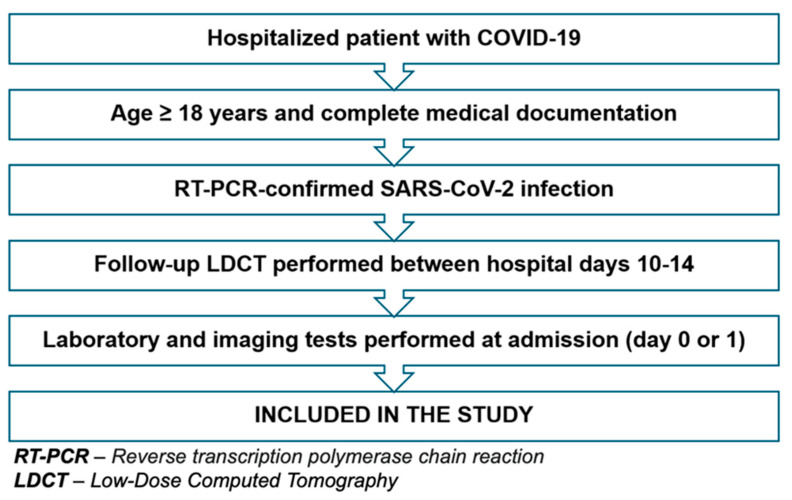
Inclusion criteria for study participation.

Study participants were informed that LDCT is an innovative imaging method that generates 10-fold lower ionizing radiation exposure compared to standard protocols. Based on the patient responses provided in these forms, inclusion criteria for the study were developed as shown in Scheme 1.

After applying the inclusion criteria, 555 patients hospitalized due to PCR-confirmed COVID-19 infection were ultimately enrolled into the study.

Patients were excluded from the study if they failed to meet any of the inclusion criteria, specifically in cases of missing follow-up chest CT (days 10–14), incomplete laboratory or clinical data, or unknown treatment outcomes (e.g., transfer to another hospital, discharge against medical advice, or death from non-COVID-19-related causes).

No formal exclusion criteria regarding comorbidities were specified in the study protocol.

### 2.4. Imaging Protocol—LDCT

The examination protocol had to strictly comply with the requirements specified by the NCCN, as presented in Table 1.

The scope of the LDCT examination covered the lungs from their apices to the costophrenic angles. The scans were conducted on deep inspiration without intravenous or oral contrast administration. In patients experiencing difficulties with breath-holding, examinations were performed during shallow, calm breathing. Subsequently, LDCT results were transferred to the Research System via the DICOM protocol, along with all reconstructions, and were registered in the system. Follow-up examinations of the patient were performed on the same CT device, employing the identical protocol as the baseline LDCT examination.

### 2.5. Image Analysis and CTSS Determination

Descriptions of LDCT examinations utilized in this project were performed by a specialist physician in radiology and diagnostic imaging with a minimum of 3 years of experience in describing chest CT (having interpreted at least 300 chest CT scans in the last 36 months), using structured reports within the Research System and adhering to current guidelines developed and approved by the Scientific Council. The Research System enabled the collection and organization of anonymized data necessary for the study, including copies of imaging data, medical information, and results of supplementary patient examinations. Additionally, it ensured independence of the database from local systems.

Report forms were completed following specific guidelines, including information on the presence or absence of contrast administration, reconstruction slice thickness of the native series, and dose-related data such as CDTI (vol), DLP, and SSDE. In the section addressing pulmonary findings, details were provided concerning the presence or absence of ground-glass opacities (GGOs), among others. Based on the LDCT results, the CTSS index was developed, allowing the assessment of pulmonary parenchymal involvement within each lobe of both lungs using the scoring presented in Table 2.

### 2.6. Statistical Methods

The collected data underwent statistical analysis in order to investigate correlations between individual features. For this purpose, the tests listed in Table 3 were utilized.

## 3. Results

### 3.1. Demographic Characteristics of the Study Population

**Figure 4:**
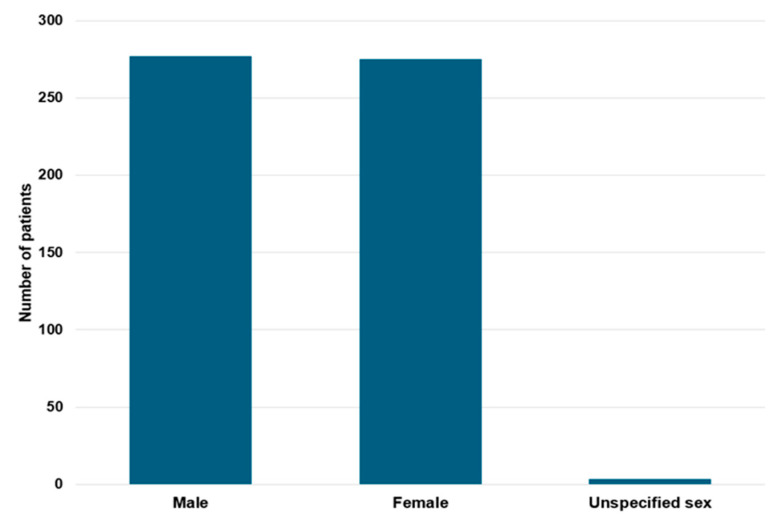
Sex distribution in the study population.

**Figure 5:**
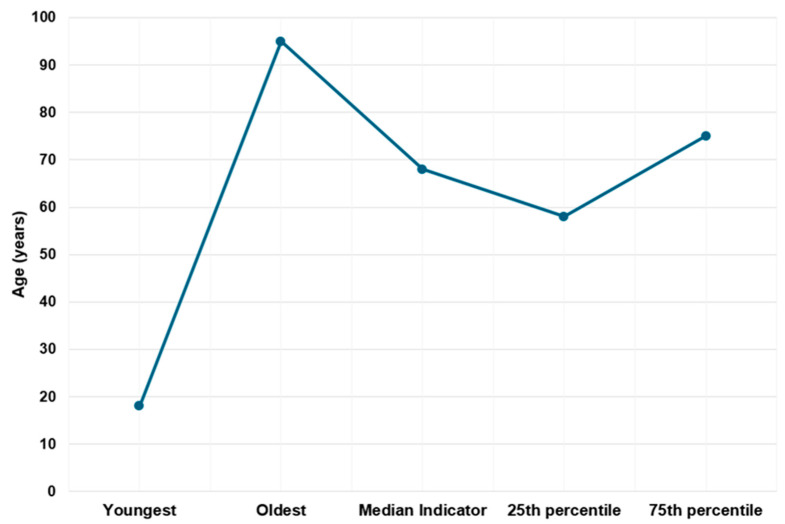
Age distribution characteristics of the study population.

Among the group of 555 patients included in the study, there were 277 men, 275 women, and 3 patients of unspecified gender, as presented in Figure 4. The youngest patient was 18 years old, while the oldest was 95 years old. The median age of patients was 68 years. Of the patients, 25% were younger than 58 years, and 75% did not exceed 75 years, indicating that the majority of the study participants were within the age range of 58–75 years. These data are illustrated in Figure 5.

### 3.2. CTSS Values in the Analyzed Group

**Figure 6:**
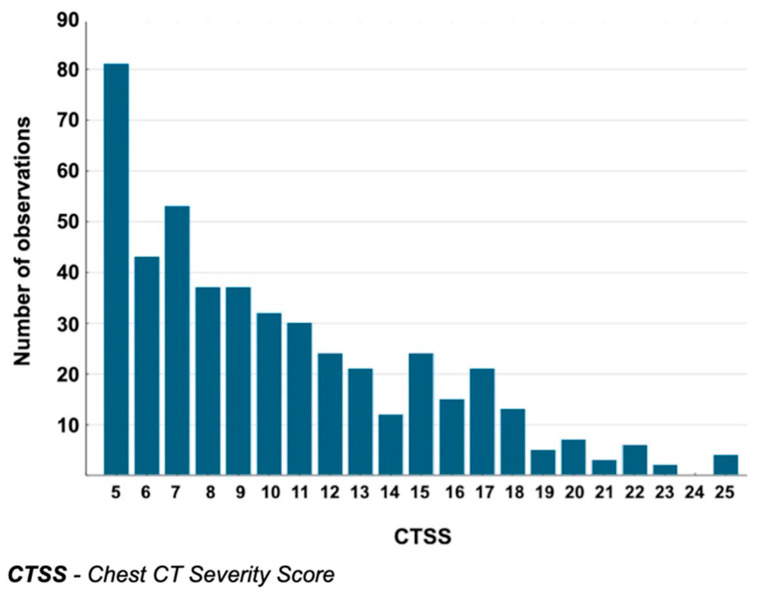
Distribution of chest CT severity score.

Based on LDCT examinations, three groups, S1 LDCT, S2 LDCT, and S3 LDCT, were established, with a corresponding CTSS designated as S1, S2, and S3, respectively. S2 LDCT and S3 LDCT examinations included the same individuals, except for a few cases where only S2 LDCT or S3 LDCT was performed; in these instances, CTSS values were incorporated into the respective analyses for S2 and S3. The results are detailed in Table 4 and Figure 6.

**Figure 7:**
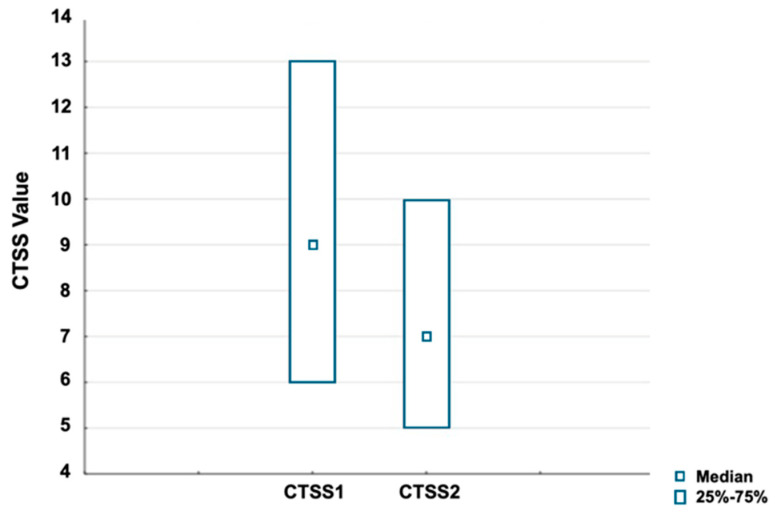
Distribution of CTSS1 and CTSS2 values, including medians and interquartile ranges.

In further statistical analyses, the S1 CTSS indicator was chosen as a reference point, as it was deemed the most representative due to the largest sample size, thereby ensuring reliability of the analysis results obtained. Furthermore, a higher statistical significance of CTSS1 was confirmed using the Wilcoxon test, as depicted in Figure 7.

### 3.3. Relationship Between CTSS and Study Endpoints

In the diagnostic analysis of the predictive value of the CTSS for key clinical endpoints —ICU admission and in-hospital mortality—low yet statistically significant AUC values were obtained. For predicting ICU admission (CTSS 13 points), the AUC was 0.616 (*p* = 0.04), and for mortality (CTSS 15 points), the AUC was 0.572 (*p* = 0.0464). These results indicate that the CTSS alone has a limited but significant ability to discriminate the risk of severe disease course. The presented graph illustrates the obtained AUC values along with confidence intervals.

**Figure 8:**
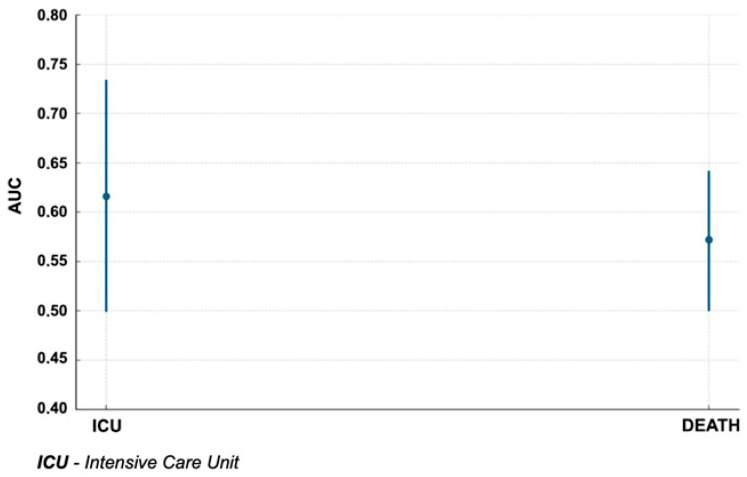
AUC values for CTSS in predicting ICU admission and in-hospital mortality (with 95% CI).

The above results are presented in Table 5 and Figure 8. After adjustment for demographic variables, age was shown to have a significant effect (*p* < 0.01), highlighting its importance as a risk factor. This association is illustrated in Table 6.

### 3.4. Type of Imaging Changes and Clinical Course

In the present study, we analyzed the influence of the localization and severity of parenchymal lesions visualized on LDCT on the duration of hospitalization and the need for ICU admission. No significant correlations were found between CTSS values and length of hospitalization (Pearson r = 0.07; *p* = 0.22; Spearman ρ = 0.01; *p* = 0.76) or length of ICU stay (Pearson r = −0.01; *p* = 0.78; Spearman ρ = 0.13; *p* = 0.06). These findings indicate no association between the CTSS and either the duration of hospitalization or ICU stay, as presented in Table 7. Additionally, a statistically significant relationship between the type of pulmonary lesions (diffuse, peripheral/subpleural, central/perihilar) and ICU hospitalization was demonstrated, confirmed by Pearson’s correlation. Bilateral lesions (n = 1091) were the most frequently observed and were also associated with the highest number of deaths (n = 113) and ICU hospitalizations (n = 222). Multiple lesions (n = 999) and disseminated lesions (n = 527) were also associated with a significant proportion of severe cases. Unilateral lesions (n = 16 in the right lung, n = 4 in the left lung) were rare and associated with a milder disease course. The mean length of hospitalization was similar across all groups, with a slightly longer stay observed in cases of left lung lesions (11.5 ± 3.53 days). These data indicated that bilateral involvement of both lungs was the most frequent finding among the studied population. The results are summarized in Table 8.

**Figure 9:**
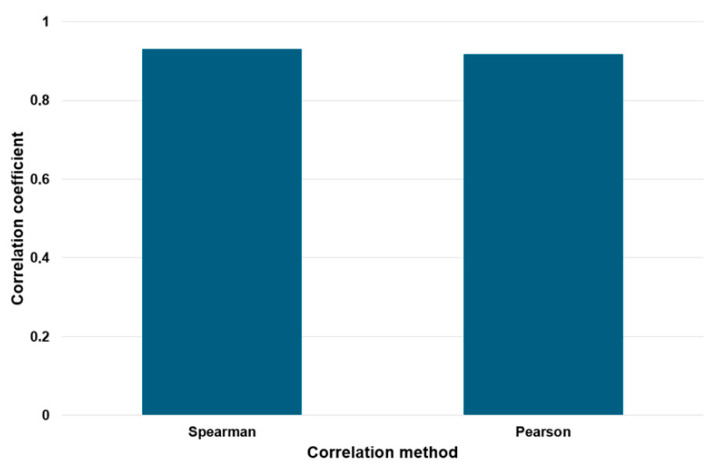
Association between age and COVID-19 severity.

A strong positive correlation was observed between patient age and the percentage of lung involvement (Spearman r = 0.93; Pearson r = 0.917; *p* < 0.01). This indicates that the proportion of affected lung parenchyma increases with age. These results are illustrated in Table 9 and Figure 9.

### 3.5. Relationship Between CTSS and Laboratory Parameters

Statistical analysis demonstrated positive correlations between CTSS values and selected laboratory parameters. A strong association was observed between lactate dehydrogenase (LDH) levels and the CTSS, as confirmed by Spearman’s correlation coefficient. Additionally, a statistically significant relationship between C-reactive protein (CRP) levels and the severity of CT changes was established. A negative correlation was also observed between lymphocyte levels and CTSS values. All aforementioned relationships are presented in Table 10.

### 3.6. Relationship Between Oxygen Saturation, Dyspnea Scale, and CTSS

Within this study, detailed statistical analysis was conducted regarding the application of oxygen therapy in patients hospitalized due to SARS-CoV-2 infection. Special attention was directed towards the analysis of relationships between selected qualitative and quantitative features and the use of oxygen therapy. For this purpose, the following parameters were assessed:

- Oxygen flow rate—a quantitative variable determining the level of oxygen support.
- Dyspnea scale with and without oxygen therapy as an indicator of subjective sensation of dyspnea among patients.
- The relationship of these parameters with other clinical and demographic features using statistical tests such as ANOVA and the Kruskal–Wallis test.

The examined relationships are presented in Table 11.

**Figure 10:**
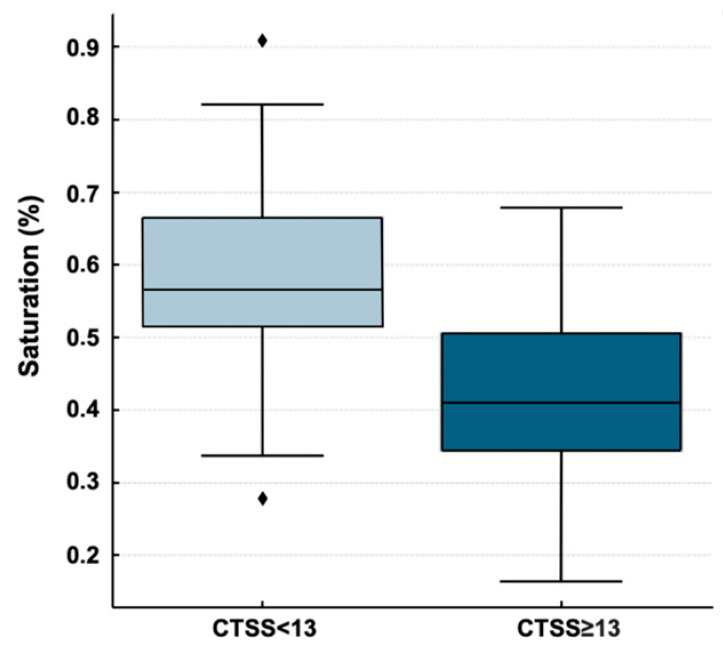
Oxygen saturation vs. CTSS.

**Figure 11:**
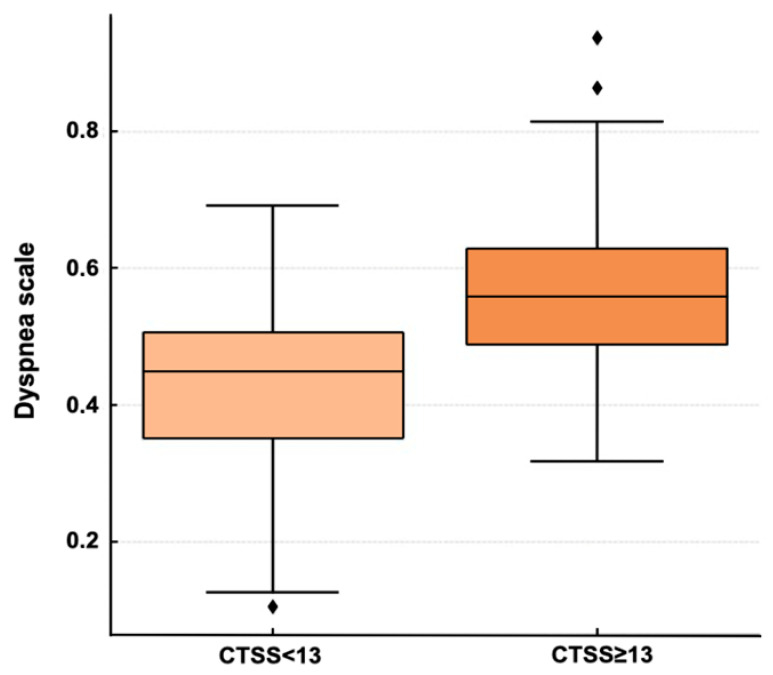
Dyspnea vs. CTSS.

Statistical analysis demonstrated significant associations between the CTSS and both subjective dyspnea assessment and oxygen saturation levels. The dyspnea scale assessed without oxygen therapy showed a positive correlation with the CTSS (r = 0.113; *p* = 0.029), as did the dyspnea scale assessed during oxygen therapy (r = 0.378; *p* = 0.030). In contrast, oxygen saturation measured at hospital admission was inversely correlated with the CTSS (r = −0.146; *p* = 0.004), indicating lower oxygen saturation values in patients with higher CTSSs. These relationships are illustrated in Table 12. Furthermore, the above-mentioned correlations were visualized—patients with a CTSS ≥ 13 exhibited significantly lower oxygen saturation levels (Figure 10) and higher dyspnea scale scores (Figure 11) compared to patients with a CTSS < 13.

## 4. Discussion

The conducted study confirmed that LDCT is an effective tool in assessing disease severity and prognosticating COVID-19 progression. Due to its high diagnostic accuracy and significantly reduced radiation exposure compared to conventional CT, LDCT may serve as a crucial component of risk stratification and patient monitoring for SARS-CoV-2 infection. In the context of COVID-19 prognosis, lower CTSSs were correlated with a decreased mortality risk even after age adjustment. Similarly, lower severity of inflammatory lung lesions assessed by the CTSS using LDCT was associated with reduced likelihood of ICU hospitalization.

### 4.1. Role of LDCT and CTSS in COVID-19 Diagnostics

LDCT enables acquisition of high-quality diagnostic images with a radiation dose reduced as much as 3.6-fold compared to standard CT, facilitating repeated examinations. Consequently, this protocol was deemed suitable for diagnostic purposes and has been recommended for clinical practice, particularly in populations requiring radiation protection [9]. Studies suggest that adequate filtering applied in low-dose protocols effectively compensates for reduced image quality [10], and LDCT itself demonstrates high sensitivity in detecting consolidations in this case, reaching 60%, and for ground-glass opacities (GGOs), 62% [11]. Given RT-PCR test limitations, such as delayed results and false-negative outcomes, computed tomography in this protocol may be a critical diagnostic tool for patients suspected of COVID-19 infection. One analysis noted that as many as 93% of clinically symptomatic patients with initially negative RT-PCR results exhibited typical inflammatory CT findings, and around 3% of all patients had false-negative initial RT-PCR tests [12]. It is worth noting that no significant correlations were found between the CTSS and length of hospitalization (Pearson r = 0.07; *p* = 0.22) or length of ICU stay (Pearson r = −0.01; *p* = 0.78). This finding suggests that although the CTSS is a useful early indicator of severe disease risk, the duration of hospitalization and ICU treatment is likely influenced by a range of other clinical factors, such as comorbidities, treatment response, and therapeutic strategies implemented across individual centers.

### 4.2. The CTSS as a Predictor of COVID-19 Severity

In this study, the CTSS was a key analytical instrument for assessing parenchymal changes within each lung lobe. This scoring system demonstrated high sensitivity (0.85) and specificity (0.86) in predicting severe COVID-19 progression [13]. Similar tendencies were also observed in our analysis, showing a strong correlation between the CTSS and critical points in the course of infection. In this study, threshold values essential for risk stratification were identified and refined, revealing that a CTSS ≥13 indicated ICU admission. CTSS values exceeding 14 points showed significant correlation with increased mortality and unfavorable clinical progression within 30 days post-admission. These analyses also confirmed the correlation between CTSSs and ICU length-of-stay [14]. Recent publications emphasize the CTSS as a strong predictor for intensive medical care requirement, independent of patient demographic characteristics [15]. According to recent findings, the CTSS may serve as a useful prognostic tool in emergency medicine, and its simplified version has been proposed to support clinical decision-making in Emergency Departments (EDs) [16]. For example, one study evaluated the CO-RADS system and CTSS scale utility for diagnosing and predicting COVID-19 progression among ED patients. This study concluded that the CTSS significantly correlated with hospitalization necessity, ICU admission, and 30-day mortality, suggesting its value in clinical decision support in ED settings [17].

A CTSS ≥15 proved a significant risk factor for death, reflected in other publications demonstrating a strong correlation between high CTSSs at admission and increased mortality among hospitalized COVID-19 patients [18]. Moreover, the semi-quantitative CTSS was also identified as an independent predictor of clinical deterioration [19].

In line with these findings, we observed significant correlations between the CTSS and several laboratory markers (Section 3.5), including CRP, LDH, IL-6, and fibrinogen. These results suggest that combining the CTSS with selected laboratory parameters may improve prognostic performance and warrants further investigation in future studies.

### 4.3. Relevance of Age and Gender in CTSS Interpretation

Our analysis revealed a significant positive correlation between age and the CTSS, suggesting more extensive lung involvement in older patients. This correlation is supported by a meta-analysis involving over 600,000 cases, demonstrating a clear increase in mortality risk with advancing age [20]. This underscores the importance of increased clinical vigilance for older patients with a high CTSS, even if initial clinical symptoms appear mild.

In younger patients, CTSS utility as a prognostic tool appears limited. Observations suggest that the predictive value significantly increases when dividing patients into groups younger and older than 65 years [21].

No significant relationship between gender and hospitalization (*p* = 0.3) was confirmed either in this analysis or in other available publications [19].

### 4.4. Role of Imaging Morphology

Single lesions within pulmonary parenchyma correlated with lower mortality, less frequent ICU admission, and shorter hospitalization (average of 9.2 days). Conversely, multiple diffuse lesions correlated with higher mortality and more frequent ICU treatment, whereas peripheral/subpleural lesions correlated with hospitalization exceeding 10 days and moderate mortality. The literature indicates that specific radiological patterns (e.g., diffuse or peripheral lesions) strongly correlate with ICU admission risk and overall disease severity [15]. Other studies also confirm that consideration of lesion type and location enhances predictive value in semi-quantitative CT evaluations [19]. A 2023 meta-analysis confirmed that lesion location and extent have prognostic significance in both acute disease progression and the risk of post-COVID pulmonary complications [22].

### 4.5. CTSS and Laboratory Markers

A significant positive correlation was noted between lesion severity assessed by the CTSS and selected laboratory parameters. The literature confirms CTSS correlation with CRP, LDH, and D-dimer levels, supporting their usefulness as markers of COVID-19 severity [23]. Some analyses also included procalcitonin as a parameter associated with the severity of radiological findings [24]. Another study noted significant correlations between the CTSS and neutrophil counts, along with negative correlations with lymphocyte percentage, aligning with this analysis and suggesting the usefulness of specific hematological markers in assessing COVID-19 progression [25]. Relationships between CRP and D-dimer levels and clinical deterioration have also been confirmed in other studies [26]. Collected data suggest that the CTSS may serve as a clinical marker correlating with inflammatory parameters, supporting rapid risk assessment of COVID-19 combined with routine admission laboratory tests.

### 4.6. The CTSS as a Predictor of Oxygen Therapy Requirements

The oxygen flow rate (in L/min) administered to patients was statistically significantly associated with other clinical parameters in all analyses (*p* < 0.05), often reaching significance at *p* < 0.001. One previous analysis demonstrated that oxygen therapy necessity—regardless of initiation timing—was associated with significantly higher pulmonary involvement assessed by the CTSS [27]. Another study divided patients into three CTSS groups (<7, 7–17, and >17), revealing significant correlations between a higher CTSS and oxygen therapy requirement [28].

### 4.7. Correlation of CTSS with Clinical Respiratory Insufficiency Symptoms

Statistical analyses revealed significant correlations between the CTSS and clinical parameters of respiratory function such as oxygen saturation and dyspnea scale. Similar correlations have been documented in other studies, where CT lung involvement positively correlated with dyspnea severity and negatively correlated with oxygenation parameters such as SpO_2_ and PaO_2_/FiO_2_ ratios [26]. Prospective studies involving hospitalized COVID-19 patients showed that a higher CTSS significantly correlated with hypoxemia and severe respiratory disease progression [29]. Other analyses have also evaluated relationships between the ROX index (combining respiratory rate and saturation) and CT lesion severity using CORADS classification. The study confirmed that early ROX index deterioration is associated with increased risk of respiratory failure and mortality, independently of radiologic severity [30]. It should be noted, however, that severe lung involvement does not always correlate with pronounced dyspnea. The phenomenon of “silent hypoxia” describes cases where patients with evident hypoxemia do not subjectively report dyspnea, emphasizing the necessity of combining imaging assessments with objective clinical evaluations, thereby confirming radiology’s high value in diagnosing and monitoring COVID-19 [31].

### 4.8. Modern Applications of Low-Dose CT and CT Severity Score in Clinical Practice and Guidelines—Development Perspectives

In recent years, artificial intelligence (AI)-based tools have gained prominence for chest CT analysis in COVID-19 patients. Machine learning models and deep neural networks achieved high effectiveness in automatic assessment of pulmonary involvement and CTSS determination. Comparative studies found that AI achieved radiologist-level performance, occasionally surpassing them in detecting subtle lesions [32].

Automated systems like COVID-Net CT, CAD4COVID, and CNN-based algorithms provide rapid, objective CTSS assessments and allow predictions of disease progression risk, ICU hospitalization need, or mortality [33]. For instance, deep learning applications in automated CT analysis achieved an AUC = 0.91 for predicting severe COVID-19 progression [34], and integrating imaging and clinical data increased classification accuracy to 93% [35].

From the perspective of routine clinical practice, automated CTSS analysis based on low-dose chest CT (LDCT) and AI tools can significantly support decision-making processes, particularly under conditions of high workload in emergency departments and intensive care units. The implementation of such systems may accelerate triage, optimize resource allocation, and facilitate disease monitoring. Although current COVID-19 management guidelines increasingly emphasize the role of CT imaging, further standardization of LDCT use and the integration of automated CTSS assessment with existing diagnostic and therapeutic algorithms remain important directions for future research and clinical practice development. The findings of our study provide additional arguments for the incorporation of these tools into future guidelines and clinical pathways for the management of acute respiratory infections.

### 4.9. Limitations

However, this study has certain limitations. It should be noted that LDCT analyses were conducted by various specialists at separate medical facilities, which introduces a risk of subjective interpretative discrepancies. Nevertheless, considerable effort was made to mitigate the impact of this variability by ensuring a standardized assessment protocol and appropriate experience among radiologists, thus enhancing the reliability of the obtained results.

Furthermore, potential variability in CT scanner models and differences in radiologist experience could have introduced inter-observer variability in CTSS scoring. Although structured reporting templates and centralized training were implemented, subtle differences in image acquisition and interpretation may have influenced the consistency of the results.

Additionally, our study cohort predominantly included older patients, with a median age of 68 years. This age distribution may limit the generalizability of CTSS-based risk stratification to younger patient populations, who were underrepresented in the present study. Further studies are needed to validate these findings in more diverse age groups.

## 5. Conclusions

A large, multicenter prospective study involving a diverse cohort of COVID-19 patients demonstrated the high clinical utility of low-dose computed tomography (LDCT) and the semi-quantitative CT severity score (CTSS) as complementary tools for risk stratification of severe disease progression. Our findings clearly confirmed the significant prognostic value of the CTSS for key clinical endpoints, such as the need for intensive care unit (ICU) admission and in-hospital mortality, with practically applicable cut-off values (CTSS ≥13 for ICU admission; CTSS ≥15 for mortality).

The strong correlation of the CTSS with clinical and laboratory parameters underscores its potential for integration with routine biochemical diagnostics, which may substantially enhance the accuracy of early risk assessment. In parallel, the low radiation dose associated with LDCT enables safe and repeatable monitoring of disease progression—an especially critical feature in dynamic and unpredictable epidemiological scenarios.

A distinctive strength of this study lies in the creation of a large, anonymized dataset of imaging and clinical data collected across multiple centers, providing a robust foundation for further research on the application of artificial intelligence (AI) algorithms in LDCT analysis and COVID-19 outcome prediction. Future work should particularly focus on the development and validation of AI-driven models to support triage processes and dynamic patient monitoring in settings of high ICU burden, especially during epidemiological crises.

Furthermore, in light of the evolving genotypic landscape of SARS-CoV-2 and the potential emergence of future viral or bacterial pandemics, future research should aim to develop and validate LDCT- and CTSS-based algorithms that can be readily implemented across diverse clinical and epidemiological contexts. The deployment of such solutions may significantly enhance the resilience of healthcare systems to future epidemiological emergencies and improve the effectiveness of crisis management.

## Figures and Tables

**Table 1:** Low-dose chest CT acquisition parameters according to NCCN guidelines.

Criterion According to NCCN	Value
Radiation dose for individuals with BMI ≤ 30	≤3 mSv
X-ray tube voltage	100–120 kVp
X-ray tube current	≤40 mAs
Detector collimation	≤1.5 mm
Reconstruction slice thickness	≤1 mm
Acquisition time	≤15 s

**Table 2:** Scoring system based on the extent of lung lobe involvement.

Score	Extent of Lobe Involvement
1	≤5%
2	5–25%
3	26–60%
4	51–75%
5	>75%

**Table 3:** Summary of statistical tests used according to variable type.

Variable Type	Test Applied	Purpose
Paired, non-parametric	Wilcoxon signed-rank test	Compare matched values before/after
Continuous, normal	Pearson’s correlation	Assess linear correlation
Continuous, non-normal	Spearman’s/Kendall’s tau	Assess monotonic association
Independent groups	Mann–Whitney U test	Compare medians between two groups
>2 group comparison	ANOVA	Compare means across multiple groups

**Table 4:** Descriptive statistics of CTSS values.

Variable		Descriptive	Statistics		
	N	Median	Q1	Q3	IQR
CTSS1	462	9	6	13	7
CTSS2	147	7	5	10	5
CTSS3	6	11	7	22	15
CTSS	470	9	6	13	7

**N**—number of observations; **Q1**—first quartile (25th percentile); **Q3**—third quartile (75th percentile); **IQR**—interquartile range (difference between Q3 and Q1).

**Table 5:** Diagnostic performance of CTSS in predicting ICU admission and in-hospital mortality.

Endpoint	CTSS Cut-Off	AUC	SE	95% CI (Lower)	95% CI (Upper)	Z	*p*-Value
ICU admission	13	0.616	0.06	0.499	0.733	1.949	0.04
Death	15	0.572	0.036	0.501	0.642	1.992	0.0464

**AUC**—area under the curve; **SE**—standard error; **95% CI**—95% confidence interval (lower/upper); **Z**—Z-statistic.

**Table 6:** Prognostic performance of CTSS for in-hospital mortality after adjustment for age.

Endpoint	CTSS (Cut-Off)	AUC (95% CI)	*p*-Value	Age Effect Adjusted
Death	15	0.572 (0.501–0.642)	0.0464	Yes

**AUC**—area under the curve; **95% CI**—95% confidence interval (lower/upper).

**Table 7:** Correlations between CTSS and hospital stay duration and ICU stay duration.

Variable	Pearson	*p*-Value (Pearson)	Spearman	*p*-Value (Spearman)
Hospital stay duration	0.07	0.22	0.01	0.76
ICU stay duration	−0.01	0.78	0.13	0.06

**Table 8:** Relationship between lesion location in LDCT and clinical outcomes, including ICU admission, mortality, and length of hospitalization.

Localization		Patients		
	Number with Symptoms	Death	ICU	Length of Hospitalization
Bilateral (both lungs)	1091	113	222	10.2 (±16.5)
Unilateral—right lung	16	0	5	8.2 (±5.11)
Unilateral—left lung	4	0	1	11.5 (±3.53)
Single lesion	110	11	29	9.2 (±5.41)
Multiple lesions	999	102	199	10.3 (±17.2)
Disseminated lesions	527	71	124	9.6 (±21.5)
Peripheral/subpleural lesions	513	39	80	10.4 (±8.5)

**Table 9:** Correlation between age and percentage of lung involvement.

Comparison	Correlation Method	Correlation Coefficient	*p*-Value
Age vs. % of lung involvement	Spearman	0.93	<0.01
Age vs. % of lung involvement	Pearson	0.917	<0.01

**Table 10:** Correlation between CTSS and selected laboratory parameters (Spearman’s rank correlation).

Laboratory Parameter	Spearman’s Coefficient	*p*-Value
CRP	0.207	<0.001
LDH	0.200	<0.001
Glucose	0.178	<0.001
WBC	0.176	<0.001
PCT	0.169	<0.001
Ferritin	0.251	<0.001
Neutrophils (%)	0.166	<0.001
Urea	0.160	<0.001
eGFR (ml/min)	−0.151	<0.001
Creatinine	0.148	<0.001
Lymphocytes (%)	−0.144	<0.001

**CRP**—C-reactive protein; **LDH**—lactate dehydrogenase; **WBCs**—white blood cells; **PCT**—procalcitonin; **eGFR**—estimated glomerular filtration rate.

**Table 11:** Statistical significance of oxygen therapy and dyspnea scores in predicting clinical outcomes.

Variable	Significance (Yes/No)	*p*-Value	Statistical Test	*p*-Value Adjusted (Age/Sex)
Oxygen flow	yes	0.001	ANOVA	0.0001
Dyspnea scale with oxygen	yes	0.035	ANOVA	0.3718
Oxygen flow	yes	0.025	ANOVA	0.0182
Oxygen flow	yes	0.0072	KW-test	No data
Dyspnea scale with oxygen	yes	<0.001	ANOVA	<0.001
Dyspnea scale without oxygen	yes	<0.001	ANOVA	<0.001
Dyspnea scale with oxygen	yes	<0.001	KW-test	0.1252
Dyspnea scale without oxygen	yes	<0.001	KW-test	0.2085
Oxygen flow	yes	<0.001	ANOVA	0.0001
Oxygen flow	yes	0.00587	KW-test	No data

**Table 12:** CTSS correlation with dyspnea severity and oxygen saturations.

Clinical Parameter	Correlation Coefficient (r)	*p*-Value
Dyspnea scale without oxygen therapy	0.113	0.029
Dyspnea scale with oxygen therapy	0.378	0.030
Oxygen saturation on admission (%)	−0.146	0.004

## Data Availability

Any data will be provided by authors if requested.
